# L’anxiété et la dépression chez les patients suivis pour microcarcinome différencié de la thyroïde

**DOI:** 10.11604/pamj.2020.35.133.12877

**Published:** 2020-04-20

**Authors:** Nassim Essabah Haraj, Siham El Aziz, Asma Chadli

**Affiliations:** 1 Service d'Endocrinologie et de Maladies Métaboliques, CHU Ibn Rochd, Casablanca, Maroc; 2 Laboratoire de Neuroscience, Faculté de Médecine et de Pharmacie de Casablanca, Université Hassan II, Casablanca, Maroc

**Keywords:** Cancer de la thyroïde, microcarcinome, irathérapie, Thyroid cancer, microcarcinoma, RAI therapy

## Abstract

**Introduction:**

Le microcarcinome de la thyroïde est un cancer de bon pronostic mais qui peut avoir un retentissement sur la qualité de vie des patients. L'objectif de l'étude est d'évaluer la dépression et l'anxiété chez les patients suivis pour microcarcinome différencié de la thyroïde et de les comparer aux autres stades du cancer.

**Méthodes:**

Étude transversale observationnelle menée entre octobre 2013 et février 2015. L'étude a inclus les patients adultes suivis pour carcinome différencié de la thyroïde. La dépression et l'anxiété ont été évaluées en utilisant deux échelles de qualité de vie, dont la traduction a été validée en arabe: le Hamilton anxiété et Hamilton dépression. Les patients ont été répartis en deux groupes, un groupe microcarcinome de la thyroïde et un groupe non microcarcinome. L'analyse des données faite par le logiciel SPSS. 16.

**Résultats:**

L'étude a concerné 37 patients suivis pour un microcarcinome différencié de la thyroïde, et un groupe de comparaison de 87 patients suivis pour autres stades de cancer différencié de la thyroïde. La qualité de vie des patients dans le groupe avec microcarcinome de la thyroïde était meilleure par rapport aux autres stades de carcinome différencié de la thyroïde. La présence de microcarcinome de la thyroïde était associée significativement à une absence d'anxiété (p = 0,042), une tendance positive a été notée sur le Hamilton dépression (mais p non significatif).

**Conclusion:**

La qualité de vie des patients dans le groupe avec microcarcinome de la thyroïde était meilleure par rapport aux autres stades de carcinome différencié de la thyroïde. Ceci pouvant être expliqué par la non agressivité du traitement (absence de traitement par l'iode, et objectif de TSH plus souple).

## Abstract (en)

**Introduction:**

Thyroid microcarcinoma is a cancer associated with good prognosis but it may affect patients’ quality of life. The purpose of this study is to assess depression and anxiety in patients treated for differentiated thyroid microcarcinoma and to compare them to patients with other cancer stages.

**Methods:**

We conducted a cross-sectional observational study between October 2013 and February 2015. The study included adult patients treated for differentiated thyroid cancer. Depression and anxiety were assessed using two quality of life scales, whose translation was validated in Arabic: Hamilton Anxiety Rating Scale and the Hamilton Depression Rating Scale. Patients were divided into two groups: thyroid microcarcinoma group and non-microcarcinoma group. Data analysis was performed using SPSS software 16.

**Results:**

The study involved 37 patients treated for differentiated thyroid microcarcinoma and a comparison group of 87 patients treated for other stages of differentiated thyroid cancer. Patients? quality of life in the thyroid microcarcinoma group was better than that of patients with other stages of differentiated thyroid cancer. Thyroid microcarcinoma was significantly associated with lack of anxiety (p=0.042), Hamilton Depression Rating Scale revealed a positive trend (p value was not significant).

**Conclusion:**

Patients' quality of life in thyroid microcarcinoma group was better than that of patients to with other stages of differentiated thyroid cancer. This can be explained by a non-aggressive treatment (absence of radioactive iodine treatment and lower TSH level).

## Introduction

Le microcarcinome différencié de la thyroïde est un cancer de bon pronostic mais qui pourrait avoir un retentissement sur la qualité de vie des patients et être source d'anxiété et dépression. De nombreuses études ont objectivé une altération de la qualité de vie chez les patients suivis pour cancer différencié de la thyroïde (CDT). Plusieurs facteurs ont été incriminés notamment le stade de la maladie, le type du traitement chirurgical, le traitement par iode radioactif, le traitement substitutif et la durée de suivi [1-6]. Une bonne relation médecin-patient basée sur la confiance, avec une évaluation psychologique tout au long du suivi permet d'améliorer la qualité de vie des patients. L'objectif de l'étude est d'évaluer la dépression et l'anxiété chez les patients suivis pour microcarcinome différencié de la thyroïde et de les comparer aux autres stades du cancer en utilisant des échelles validées de qualité de vie.

## Méthodes

Etude transversale observationnelle avec un groupe de comparaison menée entre octobre 2013 et février 2015. Le premier groupe a incluS les patients adultes suivis pour microcarcinome différencié de la thyroïde durant cette période, et dans le groupe de comparaison les patients suivis pour les autres stades de cancer. Ont été exclus de l'étude les patients ayant refusé de participer, et ceux ayant un cancer découvert il y a moins de 3 mois ou ayant un autre type de cancer. Le deuxième groupe de contrôle a inclus les patients suivis pour autre stade de cancer différencié de la thyroïde. La variable principale de l'étude est la qualité de vie, elle était évaluée par 2 échelles: Hamilton dépression et Hamilton anxiété. Les deux scores ont une traduction arabe validée [7, 8]. Pour le Hamilton dépression il comprend 21 items allant jusqu'à un maximum de 66. Un score entre 0-7 correspond à un état de santé normal, un score entre 8-13 à une dépression légère, un score entre 18-14 à une dépression modérée, un score entre 19-22 à une dépression sévère et un score ≥ 23 à une dépression très sévère [9]. En ce qui concerne le Hamilton anxiété, il comprend 14 items. Chaque item représente un groupe de symptômes différents (de 2 à 8). Chaque élément est noté sur une échelle de 0 à 4 (absent, léger, modéré, sévère, très sévère). Avec un score total allant de 0 à 56. Un score < 17 correspond à une anxiété légère, un score entre 18-24 correspond à une anxiété légère à modérée et un score ≥ 25 à une anxiété modérée à grave [10]. Les variables étudiées sont: l'âge des patients, le sexe, la durée depuis la chirurgie, la variante histologique, la taille tumorale, la réalisation d'une irathérapie, la dose thérapeutique d'iode radioactif, la présence de récidive, et la présence de métastase. Le consentement libre des patients a été obtenu avant leur inclusion, après leur avoir expliqué les objectifs de l'étude. Par ailleurs, nous avons veillé au respect de l'anonymat et à la confidentialité des données. L'analyse des données a été faite par le logiciel SPSS.16. L'analyse statistique a consisté en une analyse descriptive, et une analyse bivariée. Le seuil de signification a été fixé à 5%.

## Résultats

L'étude a concerné 37 patients suivis pour microcarcinome différencié de la thyroïde, qui ont été comparés à un groupe de 87 patients suivis pour autres stades de cancer différencié de la thyroïde. L'âge moyen des patients était de 44,7 ans, tous de sexe féminin, le nombre d'années depuis la chirurgie était de 3,95 ans, il s'agissait de microcarcinome unifocal dans 72,9 % et multifocal dans 27% des cas. La taille tumorale moyenne était de 0,86 cm. Le carcinome papillaire était prédominant dans 86,48%. L'irathérapie a été réalisée chez 40,54% des patients et la dose était ≤ 100mci. Le Tableau 1 regroupe les caractéristiques des deux groupes de patients. Concernant les paramètres de qualité de vie, la qualité de vie des patients dans le groupe avec microcarcinome de la thyroïde était meilleure par rapport aux autres stades de CDT: la dépression a été retrouvée chez 13,5 % chez les patients avec microcarcinome contre 16,1% chez les patients avec les autres stades (p = 0,715). Tandis que l'anxiété été présente chez 24,3% chez les patients avec microcancer contre 43,7% chez les patients avec les autres stades de cancer (p = 0,042). Le Tableau 2 regroupe les caractéristiques de qualité de vie dans les deux groupes de patients.

**Tableau 1:** Caractéristiques du groupe microcarcinome et des autres stades de CDT

	Microcarcinome de la thyroïde (n= 37)	Autres stades de CDT (n= 87)
Age moyen (ans)	44,70	46,29
Sexe	37F/0H	80F/7H
Nombre d’année depuis la chirurgie (années)	3,95	6,46
Taille tumoral (cm)	0,866666667	
Classification TNM	Unifocal = 27(72,9%)	TNM1 = 17 (19,5%)
	Multifocal = 10 (27%)	TNM2 = 37 (42,5%)
		TNM3= 13 (14,94%)
		TNM4 = 20 (22,98%)
Type histologique	Papillaire = 32 (86,48%)	Papillaire = 42 (48,27%)
	Vésiculaire = 0 (0%)	Vésiculaire = 6 (6,89%)
	Papil � variante vésiculaire = 5 (13,51%)	Papil � variante vésiculaire = 39 (44,82%)
Irathérapie	15 (40,54%)	75 (86,2%)
Dose supérieure à 100 mci	0 (0%)	5 (5,74%)

**Tableau 2:** Hamilton anxiété et Hamilton dépression dans les deux groupes de CDT

	Groupe micro carcinome	Groupe autres stades de CDT	P
Anxiété légère	28 (75,7%)	49 (56,3%)	0,042
Anxiété modérée à sévère	9 (24,3%)	38 (43,7%)	
Dépression normale ou légère	32 (86,5%)	73 (83,9%)	0,715
Dépression modérée à sévère	5 (13,5%)	14 (16,1%)	

## Discussion

Le concept de qualité de vie reflète un élargissement des objectifs de santé. Cette nouvelle forme d'évaluation enrichit la recherche clinique et la pratique médicale quotidienne en considérant le bien être des patients un objectif primordial dans la prise en charge. La présence d'un cancer de la thyroïde, bien qu'il s'agisse d'un cancer de bon pronostic, affecte la qualité de vie et peut être source d'anxiété et de dépression. Ceci a été confirmé par plusieurs études [1-6]. Parfois même malgré la guérison, l'excellent pronostic et le caractère peu agressif du traitement, les patients avec CDT ont une diminution de la qualité de vie qui ne peut être restaurée qu'après des années de suivis [1]. Notre étude a montré que la présence d'un microcarcinome de la thyroïde n'altère pas la qualité de vie des patients, comparativement aux autres stades de cancer de la thyroïde. Ce résultat a été retrouvé aussi dans l'étude de Taëb *et al*. [11] qui indique que les patients avec un stade de cancer plus sévère de la maladie ont des scores de qualité de vie plus bas. En effet, le microcancer de la thyroïde est considéré par certains auteurs comme une entité à part [12]. Encore plus, certains cherchent même à ne plus lui attribuer la nomination de cancer. Et des équipes ont même préconisé l'abstention chirurgicale et la surveillance des patients diagnostiqués porteurs de microcarcinome de la thyroïde [13]. Tous ces éléments nous incitent à avoir une attitude positive en annonçant le diagnostic aux patients, en leur expliquant le pronostic très favorable, ce qui permet d'éviter le retentissement sur leur bien être surtout l'anxiété. Les objectifs thérapeutiques plus souples aussi participent à avoir une meilleure qualité de vie dans ce groupe, notamment l'absence généralement d'un traitement par l'iode radioactif et les objectifs de TSHus. En effet, bien que les complications de l'irathérapie sont minimes, des effets indésirables communs peuvent être rapportés comme les nausées, les vomissements, les épigastralgies, l'altération du goût et la sialadénite, et peuvent expliquer l'altération de la qualité de vie chez les patients ayant subi un traitement par l'iode [11,14]. En ce qui concerne le taux de TSHus et son retentissement sur le bien être des patients, il reste controversé, mais certaines études ont montré que l'hyperthyroïdie infra clinique a un retentissement négatif sur le bien être [15].

## Conclusion

Notre étude montre que le groupe microcarcinome a une meilleure qualité de vie par rapport aux autres stades de cancer différencié de la thyroïde. En effet, un discours rassurant avec les patients lors du diagnostic et du suivi en expliquant le pronostic favorable de la maladie, et une prise en charge thérapeutique non agressive, permettent d'éviter aux patients dépression et anxiété.

### Etat des connaissances actuelles sur le sujet

- La présence d'un cancer de la thyroïde peut altérer la qualité de vie des patients et être source d'anxiété et de dépression.

### Contribution de notre étude à la connaissance

- La présence d'un microcarcinome différencié de la thyroïde est associée à une meilleure qualité de vie par rapport aux autres stades de cancer différencié de la thyroïde (même avec les stades de cancer où le traitement et la prise en charge est comparable au microcarcinome);
- La présence d'un microcarcinome de la thyroïde engendre moins d'anxiété.

## Conflits d’intérêts

Les auteurs ne déclarent aucun conflit d'intérêts.
